# 929. The Role of Polysubstance Use and Disposition in Burn Readmission Rates

**DOI:** 10.1093/jbcr/irag033.469

**Published:** 2026-04-06

**Authors:** Flavia E Vazquez, Nicole M Kopari

**Affiliations:** Leon S. Peters Burn Center, Fresno, CA; Leon S. Peters Burn Center, Fresno, CA

## Abstract

**Introduction:**

Readmission rates in burn patients may be a significant challenge for healthcare systems. Patients with polysubstance abuse may be at an increased risk of unplanned readmissions due to impaired adherence to follow-up care and lack of a support system. There is limited literature examining how polysubstance use and disposition planning affect readmission patterns in burn patients. We aimed to study our burn survivors and identify how polysubstance abuse and disposition may increase the rates of readmission.

**Methods:**

This was a retrospective chart review of all burn patients admitted to an ABA Verified Burn Center between January 2024 and July 2025. Patients were categorized by planned versus unplanned readmissions. Planned procedures included staged grafting and reconstructive interventions. Data was collected on demographics including reason for readmission, urine toxicology results, and discharge disposition. Polysubstance use was determined from urine toxicology results.

**Results:**

A total of 331 charts were reviewed. Thirty-one patients were readmitted to the hospital. Eleven patients (35%) were readmitted for a planned procedure. Sixteen patients (52%) had unplanned readmissions for management of infection, lack of pain control, or intolerance to wound cares. Four patients (13%) were readmitted to medical services for non-burn related reasons. 55% of the readmitted patients had a positive toxicology screens on their initial hospitalization. The most common substance of abuse was heroin, amphetamines, and polysubstance use. Fifteen patients (48%) were discharged home, seven patients (23%) were discharged home with home health, two patients (6%) were discharged to an inpatient rehabilitation center, two (6%) patients were discharged to a skilled nursing facilities, and five patients (16%) were discharged to medical respite. Two patients (6%) eloped prior to discharge.

**Conclusions:**

Despite our teams best interest in meeting all the patients’ needs prior to discharge, this review highlights the multifactorial challenges faced when discharging burn survivors. Although we did not study the number of burn patients with substance abuse, we did note that substance abuse puts patients at higher risk of readmission. These findings highlight the need for the development of early interventions, increased resources, substance abuse counseling, and additional planning aimed at our high-risk population.

**Applicability of Research to Practice:**

Larger studies are warranted for further clarification on the role of polysubstance use in readmission risk in order to reduce preventable readmissions in burn patients.

**Funding for the study:**

N/A.

